# Clinical application of high-resolution spiral CT scanning in the diagnosis of auriculotemporal and ossicle

**DOI:** 10.1186/s12880-024-01277-6

**Published:** 2024-05-09

**Authors:** Qinfang Cai, Peishan Zhang, Fengmei Xie, Zedong Zhang, Bo Tu

**Affiliations:** 1 Department of Otolaryngology, The First Clinical Medical College of Jinan University, Guangzhou, 510630 Guangdong China; 2 Department of Otolaryngology, The First Affiliated Hospital of Jinan University, Guangzhou, 510630 Guangdong China; 3 Department of Otolaryngology, The Fifth Affiliated Hospital of Southern Medical University, Guangzhou, 510900, Guangdong China

**Keywords:** High resolution spiral CT scan, Deep learning, CNN, U-net, Ossicle, Auriculotemporal

## Abstract

Precision and intelligence in evaluating the complexities of middle ear structures are required to diagnose auriculotemporal and ossicle-related diseases within otolaryngology. Due to the complexity of the anatomical details and the varied etiologies of illnesses such as trauma, chronic otitis media, and congenital anomalies, traditional diagnostic procedures may not yield accurate diagnoses. This research intends to enhance the diagnosis of diseases of the auriculotemporal region and ossicles by combining High-Resolution Spiral Computed Tomography (HRSCT) scanning with Deep Learning Techniques (DLT). This study employs a deep learning method, Convolutional Neural Network-UNet (CNN-UNet), to extract sub-pixel information from medical photos. This method equips doctors and researchers with cutting-edge resources, leading to groundbreaking discoveries and better patient healthcare. The research effort is the interaction between the CNN-UNet model and high-resolution Computed Tomography (CT) scans, automating activities including ossicle segmentation, fracture detection, and disruption cause classification, accelerating the diagnostic process and increasing clinical decision-making. The suggested HRSCT-DLT model represents the integration of high-resolution spiral CT scans with the CNN-UNet model, which has been fine-tuned to address the nuances of auriculotemporal and ossicular diseases. This novel combination improves diagnostic efficiency and our overall understanding of these intricate diseases. The results of this study highlight the promise of combining high-resolution CT scanning with the CNN-UNet model in otolaryngology, paving the way for more accurate diagnosis and more individualized treatment plans for patients experiencing auriculotemporal and ossicle-related disruptions.

## Introduction

Today, otolaryngology, the specialist area dedicated to the comprehensive evaluation of ear, nose, and throat ailments, grapples with a significant challenge [1]. It’s at a turning point in its quest for accurate and in-depth knowledge of middle ear disorders related to the auricle, temporal bone, and ossicles [2]. Due in large part to the complex anatomical components of the middle ear, diagnosing these conditions can be a challenging jigsaw puzzle [3]. Despite their value, current diagnostic approaches frequently fail to provide complete diagnoses, highlighting the pressing need for a game-changing alternative [4]. One of the most significant difficulties in otolaryngology stems from the middle ear’s complex anatomy. While conventional methods of diagnosis can be helpful in many scenarios, they often need to improve when trying to decipher issues involving the auriculotemporal region and the ossicles [5]. Due to the complexity of these disorders and the wide variety of their causes (which can range from trauma to chronic otitis media to congenital anomalies), a thorough and multi-pronged approach is required for an appropriate diagnosis [6].

The primary worry with CT scanning right now is the level of radiation exposure it exposes patients to in everyday clinical scenarios; however, with the implementation of CT technology, this problem will go away [7]. Future CT imaging evaluations of patients in all clinical contexts will be more robust and trustworthy because to a mixture of dual-energy appropriation, X-ray dose reduction, and acquisition time velocity implementation methods.

Cone beam computed tomography scans have little direct dangers. Some examples include allergic responses, nephritis, and the potential for radiation-induced cancer in the long run. There are other factors to think about, such as whether or not the patient is pregnant and the potential effects of radiation on the unborn child.

Computed tomography (CT) pictures [8] inevitably contain noise since all measurements of substance are subject to statistical error. Consequently, to improve the quality of CT images, edge-preserving denoising techniques are necessary. Noise reduction and the retention of genuine medically relevant contents are not mutually exclusive, though.It is possible to minimize or eliminate noise in CT images during the reconstruction process by employing suitable denoising filters. Consequently, denoising is necessary to enhance picture quality for better diagnosis.

Recent studies have investigated several obstacles and emerging areas in medical imaging, including the resolution of CT image noise and the creation of novel denoising algorithms to enhance image quality and diagnostic precision [9]. Several novel methodologies have been suggested for merging multimodal medical images, focusing on safeguarding data privacy and security [10]. CT scans can benefit from advanced denoising approaches, such as edge-guided filtering and collaborative feature representation networks, which have demonstrated potential in reducing noise and maintaining edge details, improving interpretability [11]. Another potentially effective method involves utilizing convolutional neural networks and fractional order total generalized variation algorithms for multimodal picture fusion and denoising in Non-Subsampled Contourlet Transform [12]. These strategies aim to address the constraints associated with particular modalities and improve the overall diagnostic efficacy of medical imaging data by using data from other imaging modalities.

This study is driven by a number of separate motivations, primarily driven by the urgent need for accurate and all-encompassing diagnostic strategies to deal with the intricacies of auriculotemporal [13] and ossicle-related disorders [14]. The first compelling force is the immediate requirement for thorough and precise diagnostic methods for auriculotemporal [13] and ossicle-related diseases [14]. These diseases frequently pose perplexing puzzles, prompting patients and medical professionals to search for better diagnostic techniques [15]. Second, there is a promising new way to deal with these diagnostic difficulties due to the development of High-Resolution Spiral Computed Tomography scanning and Deep Learning Techniques (HRSCT-DLT). This research aims to use HRSCT-DLT to advance otolaryngology by overcoming current diagnostic constraints and providing new levels of precision and insight. This research represents a paradigm change that has the potential to rethink the current system of diagnosis for disorders affecting the temporal and auricular bones. The high-resolution spiral CT scanning technique is renowned for its exceptional spatial resolution and capacity to image intricate bony structures within the temporal bone effectively. In contrast, Magnetic Resonance Imaging (MRI) offers enhanced soft tissue contrast and is frequently used to assess soft tissue pathology in the middle ear and surrounding anatomical regions. Using ionizing radiation in CT scanning raises potential concerns, particularly for pediatric patients or persons requiring recurrent imaging. MRI, as a non-ionizing modality, presents a more secure alternative. MRI can offer valuable functional information, such as dynamic imaging of the eustachian tube or evaluation of cochlear implants, which may not be attainable only by CT scanning. Temporal bone X-ray provides a rapid and economical initial assessment. Still, it may not provide the information required for a thorough review compared to CT or MRI.

The study’s central tenet is to improve diagnostic accuracy and efficiency by combining High-Resolution Spiral Computed Tomography (HRSCT) [16] scanning with Deep Learning Techniques (DLT) [17]. Incorporating the CNN-UNet deep learning method, which has been fine-tuned to perform exceptionally well in catching the finest distinctions inside medical images, is central to this groundbreaking method. This integration of cutting-edge science and medical practice gives doctors and researchers access to diagnostic technologies that promise previously unattainable levels of understanding [16]. It is clear that the union of medical imaging and deep learning has transformative potential, and this combined strategy has the potential to take patient care to new heights [18]. The primary goals of this study cover a wide range of topics. This research further advances diagnostic capabilities by investigating the complementary nature of the CNN-UNet model with high-resolution CT images [19]. Several essential tasks, such as ossicle segmentation, fracture identification, and disruption cause categorization, characterize this investigation [20]. This study aims to improve the speed and accuracy of clinical decision-making by automating these processes.

This study’s main contribution is.

- To develop a state-of-the-art diagnostic framework for automated, precise evaluation of auriculotemporal and ossicular disorders based on the HRSCT-DLT model, improving diagnostic accuracy and clinical insight in otolaryngology.
- To automate crucial diagnostic activities such as ossicle segmentation, fracture detection, and disruption cause categorization using the CNN-UNet deep learning model within the HRSCT-DLT framework for improved efficiency and accuracy in diagnosis.
- To assess the HRSCT-DLT model’s clinical effects, validate the framework’s efficacy, and pave the way for future research and advancements, this will serve as a standard for successfully incorporating cutting-edge technology into medical diagnosis.

The remainder of the article is structured as follows: Sect. 2 examines the results and limitations of several research studies in the field. In Sect. 3, the suggested methodology and its underlying architecture are described in detail. Section 4 presents the experimental results and discusses our study’s outcomes. Section 5 concludes the paper.

## Literature survey

### Segmentation of CT Scans of the Temporal Bone

Three groups of researchers have made contributions to the process of segmenting CT images of the temporal bone: Neves et al. [21], Li et al. [22], and Ke et al. [23]. To segment otologic components such as the cochlea, ossicles, facial nerve, and sigmoid sinus, Neves et al. created a CNN-based automated approach that produced very accurate results. Using promising efficacy measures, Li et al. presented a 3D-DSD Net to segment important anatomical organs. A convolutional neural network (CNN) model was developed by Ke et al. for automatic segmentation in adults and children. The model demonstrated remarkable performance for various spatial features of the temporal bone. Error analysis, misclassification, and the creation of user-friendly interfaces are all areas that still have space for development despite the progress made.

### Deep learning in ear disease diagnosis

Many researchers, including Fujima et al. [24], Wang et al. [25], Khan et al. [26], and Erolu et al. [27], have focused their attention on the utilization of deep learning in the diagnosis of a variety of ear problems. One group, Fujima et al., researched using deep-learning analysis to diagnose otosclerosis. In contrast, another group, Wang et al., developed a deep-learning technique for diagnosing middle ear problems that are persistent. The researchers Khan et al. and Erolu et al. examined the ability of artificial intelligence modelling to differentiate between individuals with chronic otitis media who had cholesteatoma and those who did not. Khan et al. revealed a novel usage of CNNs for diagnosing tympanic membrane and middle ear infections. The findings of these studies emphasize the promise of artificial intelligence in diagnosing ear diseases but also indicate the necessity of conducting additional studies in areas such as generalizability, clinical impact, and data variety.

### Diagnostic tools and techniques

Different diagnostic tools and methods are presented by Duan et al. [28], Jeevakala et al. [29], and Diwakar et al. [30] to distinguish and locate particular ear disorders. Duan et al. researched whether deep learning might be used as a diagnostic tool to differentiate between otitis media caused by OME and OM caused by PCD. Jeevakala and colleagues developed an automatic method to find and isolate the internal auditory canal (IAC) from the nerves that supply it. Diwakar et al. presented a method combining wavelet packet-based thresholding with a non-local means (NLM) filter for better edge preservation. The findings of this research demonstrate the significance of artificial intelligence in assisting radiologists in generating accurate diagnostic decisions. However, they also highlight the need for more clinical validation, generalizability testing, and optimizing interpretability.

### Perspectives from research on otosclerosis and dentistry

Asavanamuang et al. [31] suggested utilizing CBCT, or cone-beam computed tomography, to examine radiographic features associated with pre-eruptive interstitial resorption (PEIR) in teeth that have not yet erupted. The objectives of this study are to ascertain the prevalence of PEIR and its relationship to the angulation, location, and pericoronal space of teeth. Results point to the prevalence of PEIR in particular tooth orientations, highlighting the significance of CBCT monitoring, especially for molars. Silva et al. [32] described a systematic review that aims to offer evidence-based guidelines for the diagnosis and management of otosclerosis. Members of the task force receive training in knowledge synthesis techniques, and they evaluate literature to provide recommendations on treatment (such as surgery, medication, hearing aids, and implantable devices) and diagnosis (including audiologic and radiologic) based on predetermined parameters.

The study developed a state-of-the-art diagnostic framework for automated, exact evaluation of auriculotemporal and ossicular abnormalities using the HRSCT-DLT model, enhancing otolaryngology diagnostic accuracy and clinical insight. Optimize diagnosis efficiency and accuracy by automating ossicle segmentation, fracture identification, and disruption cause categorization using the CNN-UNet deep learning model in the HRSCT-DLT framework. This will set a precedent for effectively integrating cutting-edge technology into medical diagnostics by assessing the HRSCT-DLT model’s clinical impacts, validating the framework, and enabling future research and developments.

Medical imaging aims to detect and track healthy and diseased bodily structures and functions by creating three-dimensional models of individual organs and tissues. Various medical imaging modalities are utilized for this aim, including X-ray, CT, PET, MRI, digital mammography, diagnostic sonography, and many more. Cardiovascular diseases, cancer of various tissues, neurological problems, congenital heart conditions, complications in the abdomen, complicated broken bones, and many other significant illnesses can be better diagnosed with the use of these cutting-edge medical imaging tools. Any kind of imaging has its advantages and disadvantages. Two main approaches exist for temporal skeleton computed tomography (CT) accumulation: a dual intake with independent bilateral axial and panoramic scans or a single axially recorded volume with coronal and if desired, sagittal reorganizes applied to the longitudinal source data. While contrast medication can be useful in certain cases, including when looking for otomastoiditis issues, vascular tumors, or vascular anomalies, it is usually not needed for routine evaluations of coalescence, mastectomy air cell death, or hearing loss. Because of CT’s superior contrast compared to traditional hypocycloidal tomography, traumatic ossicular disturbances may now be seen. Additionally, congenital anomalies of the stapes’s framework can be better seen.

The proposed HRSCT-DLT model symbolizes a harmonious merger of high-resolution spiral CT scanning and the CNN-UNet model. This union is designed to address the nuances of auriculotemporal and ossicular disorders. It is not only a shortening of the diagnostic procedure; it marks an ascension in our grasp of these delicate situations, defining the boundary of medical imaging and diagnostics. This research intends to help doctors make more accurate diagnoses by highlighting the possibilities of combining high-resolution CT scans [33] with the CNN [34] and UNet [19] models in otolaryngology. In addition, this method facilitates the development of patient-specific treatment plans for auriculotemporal and ossicular disorders. The ultimate goal is for this game-changing strategy to transfer to better patient outcomes and a higher general level of care. The research has the potential to herald a new era of precision and quality in otolaryngology through its careful path of discovery, customization, and application. The study developed a state-of-the-art diagnostic framework for automated, exact evaluation of auriculotemporal and ossicular abnormalities using the HRSCT-DLT model, enhancing otolaryngology diagnostic accuracy and clinical insight. Optimize diagnosis efficiency and accuracy by automating ossicle segmentation, fracture identification, and disruption cause categorization using the CNN-UNet deep learning model in the HRSCT-DLT framework. This will set a precedent for effectively integrating cutting-edge technology into medical diagnostics by assessing the HRSCT-DLT model’s clinical impacts, validating the framework, and enabling future research and developments (Table 1).

**Table 1:** Literature survey

Author	Method	Application	Limitation
Neves et al. [21]	CNN-based automated system for segmenting CT scans	Automatic temporal bone CT segmentation using CNN. The models learned to segment the cochlea, its ossification facial nerve, and sigmoid sinus.	Error and misclassification analysis, as well as the creation of intuitive user interfaces, still have space for development.
Li et al. [22]	3D-DSD Net	The highly connected network uses 3D multi-pooling feature fusion. Dice factor, precision, sensibility, and Hausdorff distance evaluate efficacy.	3D-DSD Net’s generalizability, clinical integration, and error analysis need more research.
Fujima et al. [24]	DL analysis for identifying otosclerosis	DL systems like AlexNet, and ResNet to evaluate their examination data and develop a diagnostic model.	Need more attention on in generalizability, error assessment, clinical impact, and data variety.
Ke et al. [23]	CNN based auto segmentation	The impetus was automatic temporal bone CT segmentation in adults and children.	Maintain limits in real-time clinical circumstances, produce accurate predictions, and expand the dataset to include individuals with more characteristics and diseases.
Wang et al. [25]	Diagnose persistent middle ear diseases using DL.	MESIC used a “region of interest” (ROI) area search network and a classification network to provide reliable diagnoses.	Not efficient and trustworthy
Khan et al. [26]	CNN-Medical imaging	An innovative use of CNNs, including state-of-the-art models like DenseNet, to automatically identify TM and ME infections in medical imaging.	Need attention on clinical validity, data diversity, and interpretability
Erolu et al. [27]	AI –CT scans	AI modeling was utilized to determine if CT scans of chronic otitis media (COM) patients could distinguish cholesteatoma from non-cholesteatoma.	Need AI for correct diagnosis Acute cholesteatoma
Duan et al. [28]	DL-PCD screening	Deep learning’s capacity to distinguish OME-related otitis media (OM) from PCD-related OM.	Limited accuracy and reliability
Jeevakala et al. [29]	Automated method for IAC location and nerve separation	The Mask R-CNN and U-Net-powered approach located and segmented the IAC and nerves, studies indicated.	Computational complexity

This literature review investigates otolaryngology and otologic imaging analysis, focusing on applying deep learning approaches, particularly CNNs. CT scans of the temporal bones have been segmented automatically using CNNs, with impressive results in accuracy and overlap with the human ground truth. The studies stress the significance of user-centred design, mistake detection and correction, clinical validation, data variance, and interpretability. Otosclerosis, chronic middle ear illnesses, tympanic membrane and middle ear infections, and differentiating between comorbidities caused by OME and PCD are all successfully diagnosed using deep learning algorithms. The study suggests combining high-resolution spiral CT scanning with deep learning techniques (HRSCT-DLT) for effective and trustworthy diagnosis of auriculotemporal and ossicle-related disorders.

## Propoced system model

The proposed research intends to change otolaryngology by increasing the accuracy and efficiency of diagnostic procedures by combining High-Resolution Spiral Computed Tomography scanning with Deep Learning Techniques (HRSCT-DLT). The CNN-UNet deep learning model is at the heart of this groundbreaking method, and it has been fine-tuned to excel in capturing minute details in medical photos. This integration of cutting-edge science and medical practice gives doctors and researchers access to diagnostic technologies that promise previously unattainable levels of understanding.

With High-Resolution Spiral Computed Tomography scanning and the CNN-UNet deep learning model, the HRSCT-DLT model helps doctors and scientists capture sensitive data in medical images. This method can improve the treatment of patients and diagnostic time by automating ossicle categorization, fracture diagnosis, and disruption cause categorization. Automation of clinical decision-making improves diagnosis accuracy and reduces medical staff workload. Cutting-edge medical imaging and diagnostics tools like the HRSCT-DLT model help clinicians make accurate diagnoses and customize patient care.

**Fig. 1:**
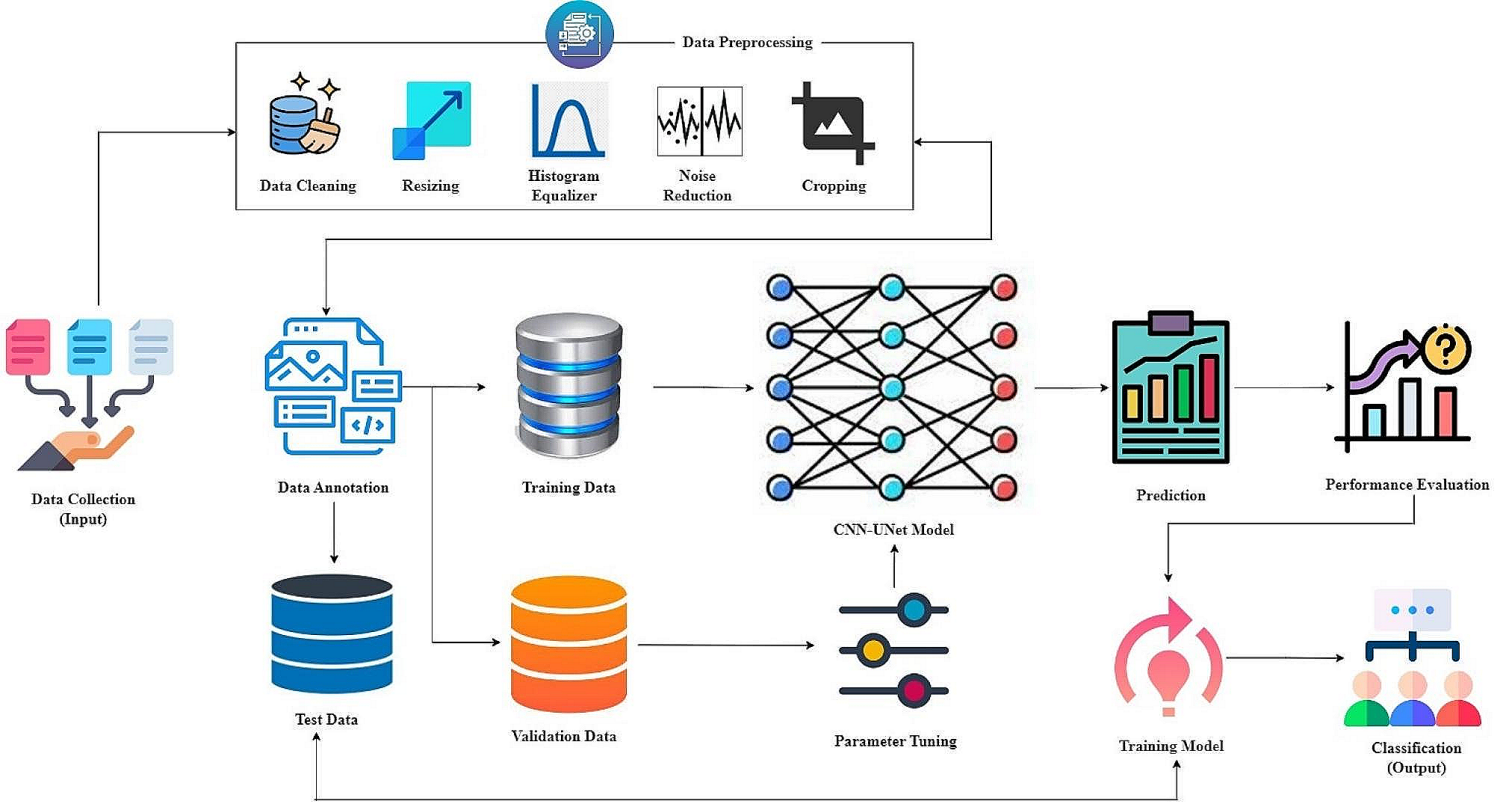
HRSCT-DLT system model

Figure 1 portrays the system architecture of the suggested HRSCT-DLT approach.

Figure 1 shows how the HRSCT-DLT plans to revolutionize otolaryngology by making diagnostics more precise and faster. With a major focus on auriculotemporal and ossicular illnesses, this study will develop a database of CT scans of the temporal bones. The collection contains detailed information about the middle ear’s anatomy, acquired using HRSCT imaging. The accuracy of the deep learning model relies heavily on the annotations provided by medical professionals. These experts separate relevant data into its component parts, such as ossicles, fracture sites, and disruption triggers. When working with medical picture data, data preparation is absolutely necessary. Prior to training your Convolutional Neural Network-UNet (CNN-UNet) model, you must conduct data preprocessing on your High-Resolution Spiral Computed Tomography (HRSCT) scans using deep learning techniques. The fundamental objective of these rigorous preparation steps is to meticulously get your data ready for the next CNN-UNet model training. The CNN-UNet model excels at precise segmentation in medical images, which are particularly useful for depicting the intricate anatomy and subtle disorders affecting the middle ear. For the best results in picture segmentation, try using the CNN-UNet technique, which combines CNN with U-Net. When using high-resolution CT data to segment anatomical components in the middle ear, the HRSCT-DLT model relies heavily on CNN-UNet. Because of its well-calibrated convolutional layers, the CNN-UNet model is able to pick up on the tiniest of anatomical details.

### Data collection and preparation

A database of CT scans of temporal bones will be created for this study, with a primary focus on auriculotemporal and ossicular disorders. Detailed anatomical information regarding the middle ear may be found in the dataset, which was gathered via HRSCT imaging. Congenital abnormalities, concussions, and recurrent ear infections are just some of the many medical conditions addressed. The purpose of this comprehensive dataset is to simplify the field of otolaryngology by illuminating all aspects of auriculotemporal and ossicle-related illnesses. Since it encompasses such a large and comprehensive dataset, this study is ideal for tackling the complexity and nuances that drive the discipline of otolaryngology since it offers a bird’s-eye view of auriculotemporal and ossicle-related problems.

### Data annotation

Medical experts’ annotations are crucial to the performance of the deep learning model. These specialists isolate and define data subsets of interest, such as ossicles, fracture locations, and disruption triggers. The ground truth labels provided by these annotations are crucial to the success of the deep learning procedure. The CNN-UNet model requires these labels for thorough training and validation. Using these comparisons, the deep learning model can be trained to become a reliable diagnostic tool in the context of the study.

### Data preprocessing

Data preprocessing is essential in preparing your data, especially in medical image analysis. Auriculotemporal and ossicular illnesses will be the focus of this study’s temporal bone CT scan database. The collection contains HRSCT-imaged middle ear anatomy. Congenital defects, concussions, and recurring ear infections are among the medical issues treated. This comprehensive dataset simplifies otolaryngology by revealing all auriculotemporal and ossicle-related diseases. This study provides a s-eye view of auriculotemporal and ossicle-related issues. It is perfect for confronting the complexity and nuances that drive otolaryngology due to its big and thorough dataset. Using deep learning techniques with High-Resolution Spiral Computed Tomography (HRSCT) scans, you must first perform some preprocessing to get the data into shape for your Convolutional Neural Network-UNet (CNN-UNet) model. Consider the following pre-analysis steps for your data:

(i) ***Data Cleaning***: Noise in high-resolution medical images like CT scans can have many causes, including human error, faulty equipment, and the surrounding environment. Diagnostic accuracy and image analysis precision are both susceptible to noise. Gaussian and other noise reduction filters can reduce background noise without losing valuable diagnostic information. Images obtained from CT scanners can benefit from these filters’ improved clarity and resolution.
(ii) ***Image Resizing***: Reduce the images’ size until they fit your CNN-UNet’s criteria. Computing-intensive high-resolution scans can benefit from scaling, which also helps to standardize the data. The new pixel values in the scaled image are determined using a weighted average of surrounding pixels from the original image due to the resampling technique of bilinear interpolation. This method lowers the image’s size without degrading its overall quality.
(iii) ***Histogram Equalization***: This method can improve the contrast of medical images by shifting the relative brightness of individual pixels. Enhancing the clarity of finer details is one area where it can be beneficial. High-resolution CT scan images can improve their contrast and overall visual quality with the help of histogram equalization. Contrast Limited Adaptive Histogram Equalization (CLAHE) is a widespread method for histogram equalization. CLAHE improves classic histogram equalization since it accounts for regional differences within an image, making it ideal for diagnostic tools like CT scans.
(iv) ***Noise Reduction***: Noise in medical images might degrade the quality of any subsequent analysis. Reduce noise with methods like median filtering and Gaussian smoothing. Non-local means (NLM) Denoising is an efficient method for reducing noise in high-resolution CT scan pictures. The NLM approach is frequently utilized in the medical imaging processing industry to reduce noise while maintaining image features.
(v) ***Cropping***: Cropping images to isolate the area of interest can simplify processing by removing extraneous data. Manual or semi-automated region-of-interest (ROI) selection is typical for cropping high-resolution CT scan pictures. A radiologist or other medical professional analyzes the image to pinpoint the location of any pathology or anatomical structures of interest.

The primary goal of such stringent preprocessing processes is to methodically prepare your data, setting a firm groundwork for the upcoming training of the CNN-UNet model. Ossicle segmentation, fracture identification, and disruption cause categorization are complex and diverse procedures requiring meticulous data preparation in auriculotemporal and ossicle-related disorders. These steps in preparation guarantee that the data is polished to perfection, ready to provide the best training and validation results possible for the model.

### CNN-UNet model development

The proposed methodology centres on the creation and refinement of the CNN-UNet deep learning model, which is essential to the diagnostic framework of the research. Medical images, such as those showing the complex anatomy and subtle diseases of the middle ear, are ideal candidates for the CNN-UNet model’s exact segmentation. The CNN-UNet model’s greatest asset is its well-calibrated convolutional layers, which allow it to catch even the minutest anatomical information. Due to its complexity and relative fragility, medical imaging analysis of the middle ear is of the utmost relevance. The CT scans can reveal even the tiniest of abnormalities, but the convolutional layers were built with sensitivity to ensure nothing was overlooked. CNN-UNet’s training phase is rigorous to ensure the model is up to the task of recognizing and segmenting important structures inside CT scans, and this step is crucial to the model’s eventual success. The use of the meticulously documented dataset facilitates this procedure. During training, the model absorbs information from the dataset to improve its knowledge and ability to identify target areas inside images. The model improves at detecting and outlining critical structures through this iterative learning process, making it more useful for precise diagnosis.

The CNN-UNet model is a sophisticated deep-learning technique capable of precisely delineating ossicles, which are small and fragile bones located in the middle ear. This process dramatically aids in the detection and examination of anomalies or disorders. Additionally, it can accurately identify fractures in the temporal bone, which is vital for auditory function and overall well-being. The proposed model employs deep learning techniques to evaluate CT images and effectively identify regions that suggest fractures. It enables doctors to focus on these specific locations for subsequent assessment. Additionally, it aids in categorizing the reasons for disruption in the temporal bone, which can arise from factors such as trauma, infection, or congenital anomalies. This information assists healthcare professionals in making precise diagnoses and developing personalized treatment strategies. The incorporation of the CNN-UNet model into high-resolution CT images improves the effectiveness and precision of diagnostic procedures in the field of otolaryngology. This integration automates several activities: segmentation, fracture identification, and categorizing disruption causes. This novel methodology enables healthcare professionals to make well-informed choices that maximize patient results.

#### Convolutional neural network model

The visual data processing and analysis tasks that CNNs, a subset of deep neural networks, excel at include image classification, segmentation, and object detection. CNNs are excellent at jobs involving patterns, such as those observed in medical imaging, since they are made up of layers that automatically acquire features from the data. CNNs play the role of feature extractors in the HRSCT-DLT framework. They perform an in-depth analysis of the provided CT scans, deciphering essential patterns and structures that are fundamental to grasping the complex anatomy of the middle ear. Edges, textures, forms, and spatial interactions between image components are all potential candidates for such patterns. Consider the CT picture $X$ to be the input. Convolutional neural networks (CNNs) examine $X$ as an input image and extract features $F$ that describe salient aspects of the image. This operation can be mathematically expressed by Eq. (1).

1 $$F=CNN\left(X\right)$$

The extracted features are denoted by $F$, while CNN indicates the Convolutional Neural Network. Convolutional layers are the building blocks of CNNs, and they use a set of learnable filters or kernels to process the input image. To make things easier, this study refers to the input image ($X$) and the convolution procedure ($*$). The filters are portrayed as $K$ (kernels), while the output feature maps are denoted as $Y$. The mathematical expression for this convolution is shown in Eq. (2).

2 $$Y=X * K$$

Here, $X$ is the input picture, $Y$ are the feature maps, and $K$ are the convolutional kernels. Sliding the kernels about the input image systematically is what the convolutional process does to pick up on local patterns like edges and corners. It is common practice to downsample the data using pooling layers following the convolutional layers. For example, max-pooling takes a small area within each feature map and picks its maximum value. Equation (3) is a symbolic illustration of the pooling process.

3 $$Y=Max-Pool\left(X\right)$$

Here, $Y$ is the feature map after downsampling, and Max-Pool is the maximum pooling operation. Each convolutional layer generates feature maps, which represent various picture features. These feature maps stand in for data abstractions. Equation (4) provides an algebraic model for a layer with $N$ feature mappings.

4 $$F=({F}_{1},{F}_{2},\dots ,{F}_{N})$$

An ${i}^{th}$ the variable denotes the feature map ${F}_{i}$. The HRSCT-DLT model uses convolutional neural networks (CNNs) to segment middle ear anatomy. The CNN’s learned characteristics form the basis for the segmentation procedure. As shown in Eq. (5), the input CT image $X$ generates the segmented output $S$.

5 $$S=Segmentation-CNN\left(X\right)$$

Where $S$ is the image after segmentation, and the convolutional neural network (CNN) employed for segmentation is denoted here as $Segmentation-CNN$. Training a CNN takes a lot of time and labelled data. Training the HRSCT-DLT model requires the use of labelled data. To make things easier to understand, we’ll refer to the annotated dataset as $D=({X}_{i},{Y}_{i})$, where ${X}_{i} ,$is the input CT image, and ${Y}_{i}$, are the ground truth labels identifying the location of structures. Using a loss function (typically represented by the letter L) during training is common practice to decrease the gap between the model’s predictions and the truth. This method fine-tunes the model to generate the correct segmentations, as indicated in Eq. (6).

6 $$\theta *=arg\,\text{min}\,\theta \frac{1}{\left|D\right|} {\sum }_{(X,Y \in D)}^{ }L(Segmentation-CNN\left(X\right), Y)$$

Here, $\theta$ stands for the original model parameters, L for the loss function, and * for the optimal set of values. The CNN can make inferences about novel, unseen CT images following training. It accepts an image as input and produces a segmented output focusing on specific features (such as ossicles or fractures) inside that picture. The segmented output produced by CNN supports healthcare practitioners in making diagnostic decisions. It expedites clinical care by improving accuracy and efficiency through automated examination of critical anatomical structures and diseases.

#### CNN-UNet algorithm in HRSCT-DLT framework

The CNN-UNet strategy is a CNN and U-Net hybrid optimized for image segmentation. CNN-UNet is critical in the HRSCT-DLT model for segmenting middle ear anatomical structures from high-resolution CT data. The U-shaped design of the U-Net design is a defining feature of the encoder and decoder. The encoder downsamples the input image to capture relevant components; the decoder then upsamples these features to produce the segmentation map. The CNN-UNet starts by operating as a feature extractor. As input, it accepts high-resolution CT images, such as those of the temporal bone. The U-Net uses convolutional layers to process the input image in the encoder, which is a convolutional neural network. These layers identify specific details, structures, and patterns in the image. Let’s call this first step in the process “feature extraction,” and let’s refer to the input image as ${I}_{in}$, in Eq. (7).

7 $${F}_{cnn}=CNN\left({I}_{in}\right)$$

Here, ${F}_{cnn}$, stands for CNN’s gleaned feature maps. These feature maps represent small-scale variations in the input image’s overall structure, colour, and texture. The image’s spatial dimensions are decreased while the encoder’s feature channel count rises. The encoder’s successive layers can record increasingly abstract characteristics. Convolutional layers using max-pooling or strided convolutions accomplish this. Let’s use Eq. (8) to represent the encoding procedure.

8 $${E}_{cnn}=Encoder\left({F}_{cnn}\right)$$

High-level feature maps are encoded and stored in the variable ${E}_{cnn}$. The model takes the most essential features from the U-Net’s bottleneck and keeps their high-level representation. Equation (9) is a graphical representation of the bottleneck property.

9 $${B}_{cnn}=Bottleneck\left({E}_{cnn}\right)$$

The U-Net’s decoder starts upsampling the bottleneck’s high-level characteristics. Upsampling raises the number of spatial dimensions, enabling the identification of features inside an image that may be described using the formula (10).

10 $${D}_{cnn}=Decoder\left({B}_{cnn}\right)$$

Where, ${D}_{cnn},$ is a variable that stores the decoded feature maps. The presence of skip connections is an essential part of the U-Net design. These bridges open the encoder’s data to the decoder on various levels. Equation (11) depicts the importance of skip connections in preventing the loss of fine-grained information during the encoding and decoding processes.

11 $${S}_{cnn}=SkipConnections({E}_{cnn},{D}_{cnn})$$

The ${S}_{cnn}$, variable represents enriched feature maps achieved by skip connections. The decoder creates the final segmentation map as the features are upsampled with skip connections. This map emphasizes the regions that are intriguing within the supplied image. Equation (12) is a valuable representation of the segmentation procedure.

12 $${S}_{output}=Segmentation({D}_{cnn},{S}_{cnn})$$

Here, $${S}_{output}$$​, represents the segmented output, a map highlighting regions of interest, such as ossicles or fractures. The CNN-UNet model is trained using annotated datasets that contain input CT images ($${I}_{in}$$​) and ground truth labels for segmentation ($$GT)$$. At the heart of the training process is a loss function (usually a pixel-wise cross-entropy loss or a dice loss), whose goal is to reduce the discrepancy between the model’s forecasts and the ground truth labels (Eq. (13).

As a map emphasizing regions of interest like ossicles or fractures, ${S}_{output}$, depicts the segmented output. Input CT images (${I}_{in}$) and ground truth labels for segmentation (GT) are used to train the CNN-UNet model from annotated datasets. Preparing the model entails optimizing its parameters with a loss function (usually a pixel-wise cross-entropy loss or a dice loss) to reduce the discrepancy between the model’s predictions and the ground truth labels, as shown in Eq. (13).

13 $$Loss=Loss({S}_{output},GT)$$

The model is fine-tuned through this optimization process to produce reliable segmentations. After training the CNN-UNet, it can infer information from fresh CT scans. It accepts an image as input and produces a segmented result with relevant parts. By applying Eq. (14) to an image input (${I}_{in}$), it has a segmented image output (${S}_{output}$).

14 $${S}_{output}=Inference\left({I}_{in}\right)$$

The CNN-UNet model provides a segmented output (Soutput) sound for medical diagnosis. It expedites clinical care by improving accuracy and efficiency through automated examination of critical anatomical structures and diseases. Algorithm 1 (Table 2) exemplifies how this comprehensive pipeline uses convolutional neural networks and the U-Net architecture to improve the HRSCT-DLT model’s picture segmentation and diagnostic capabilities.

**Table 2:** Algorithm 1 - HRSCT-DLT model

Algorithm 1: HRSCT-DLT Model
*Begin*
***Step 1***: *Define hyperparameters* *input_shape = (img_height, img_width, img_channels) # Define image dimensions* *n_classes = num_classes # Define the number of segmentation classes* *learning_rate = 0.001* *batch_size = 32* *epochs = 50* ***Step 2***: *Define a function to build the CNN-UNet model* *function build_cnn_unet(input_shape, n_classes)* *inputs = Input(input_shape)* *# Encoding Path* *conv1 = Convl2D(64, 3, activation=’relu’, padding=’same’)(inputs)* *conv1 = Convl2D(64, 3, activation=’relu’, padding=’same’)(conv1)* *pool1 = MaxPooling2D(pool_size=(2, 2))(conv1)* *# Including more encoding layers* *# Decoding Path* *up6 = UpSampling2D(size=(2, 2))(conv6)* *up6 = Convl2D(64, 2, activation=’relu’, padding=’same’)(up6)* *merge6 = Concatenate(axis = 3)([conv3, up6])* *conv6 = Convl2D(64, 3, activation=’relu’, padding=’same’)(merge6)* *conv6 = Convl2D(64, 3, activation=’relu’, padding=’same’)(conv6)* *# Including more decoding layers…* *# Output Layer* *out = Convl2D(n_classes, 1, activation=’softmax’)(conv10)* *return Model(inputs = inputs, outputs = out)* ***Step 3***: *Load and preprocess your dataset* *X_train, Y_train = load_and_preprocess_data(data_path)* *X_train, X_val, Y_train, Y_val = split_train_and_validation_data(X_train, Y_train, validation_ratio)* ***Step 4***: *Build and compile the model* *model = build_cnn_unet(input_shape, n_classes)* *model.compile(optimizer = Adam(learning_rate)*, *loss=’categorical_crossentropy’, metrics=[‘accuracy’])* ***Step 5***: *Train the model and save* *model.fit(X_train, Y_train, batch_size = batch_size, epochs = epochs, validation_data=(X_val, Y_val))* *model.save(‘HRSCT-DLT_model.h5’)* ***Step 6***: *Perform segmentation on the test dataset* *function segment_new_images(new_images, model)* *predictions = model.predict(new_images)* *return predictions* ***Step 7***: *End*

A Convolutional Neural Network - U-Net (CNN-UNet) model and the steps required to construct, train, and employ it for semantic image segmentation. Medical image analysis frequently involves segmenting images into various classes, such as segmenting anatomical components in high-resolution CT scans. Image size, segmentation class count, learning rate, batch size, and training iterations are all important hyperparameters to tweak. The CNN-UNet model consists of an input layer, a hidden layer for decoding, and an output layer. The algorithm reads the training data, cleans it up, creates an Adam optimizer, loss function, and evaluation measure (in this case, accuracy), trains the model for a given number of iterations, and stores the result. For applying the trained model to the segmentation of brand-new, unseen images, the method additionally defines the function segment_new_images. They are combining the capabilities of CNNs for feature extraction with those of the U-Net architecture for image segmentation results in the CNN-UNet architecture. It is an essential part of the HRSCT-DLT model for accurate and automated segmentation and detection of auriculotemporal and ossicle-related disorders in the middle ear because of its ability to capture delicate anatomical details inside high-resolution CT scans.

The proposed methodology relies heavily on a deep learning model called CNN-UNet that was built from the ground up to meet the specific challenges of this research. Owing to its design, fine-tuned convolutional layers, and rigorous training on the annotated dataset, it can adequately identify and segment essential structures inside temporal bone CT scans, improving precision and insight into otolaryngology. This concept has the potential to change otolaryngology (ENT) diagnostics and bring about significant improvements in patient treatment.

In this section, we outline the methodology and framework that will enable High-Resolution Spiral Computed Tomography scanning and Deep Learning Techniques (HRSCT-DLT), and especially the CNN-UNet deep learning technique, to revolutionize otolaryngology. This tool was developed to aid otolaryngologists in their work by giving them a synopsis of all the disorders that might affect the auricle, temporal bone, and ossicles. This novel approach has the potential to revolutionize the ENT industry because of the architecture and training technique of the CNN-UNet model.

Compared to more traditional forms of chest imaging, high-resolution computed tomography (HRCT) allows for a clearer view of the lungs’ complex structures and the detection of subtle disease changes. By excluding variations caused by gravity or dependent atelectasis, upright HRCT imaging is helpful for individuals with basal illness.

Methodology chosen for the purpose of identifying and selecting studies that will further improve diagnostic skills by exploring how high-resolution CT images complement the CNN-UNet model. Obssicle segmentation, fracture recognition, and disruption cause categorization are some of the important tasks that this inquiry focuses on.

## Experimental results and analysis

### Setup

Due to its high diagnostic accuracy, the HRSCT-DLT Dataset relies heavily on HRSCT imaging of the temporal bone. Traumatic injuries, chronic otitis media, congenital disabilities, and auriculotemporal and ossicle-related illnesses constitute only a few of the many middle ear conditions included in the dataset [35, 36]. This study uses a randomized stratified split depending on the prevalence of various illnesses to separate the dataset into training, test and validation sets of 80%, 10% and 10%, respectively. The study uses standard image segmentation measures like Dice Coefficient, Recall, Precision, F1 Score, Root Mean Squared Error (RMSE), Mean Absolute Error (MAE), Hausdorff Distance, and Intersection over Union (IoU) to assess CNN-UNet’s performance. We evaluate the proposed model’s efficacy and utility inside the HRSCT-DLT framework by contrasting it against several other deep learning models, such as CNN-GoogLeNet, CNN-DenseNet, CNN-ResNet, and Mask-R-CNN-UNet.

A detailed description of the tests, together with the results and data obtained, is provided in this section. The paper describes the experimental framework that was created, the dataset that was utilized, and the method used to partition the dataset into training and testing sets. Also included are comparisons to other models of its kind and an explanation of the criteria used to assess the HRSCT-DT model. Several metrics pertinent to medical image segmentation, including accuracy, recall, F1 score, dice coefficient, IoU and error measures like RMSE and MAE, demonstrate outstanding performance by the HRSCT-DT model. It delves deep into the ramifications of the HRSCT-DT model’s effectiveness for medical image analysis, specifically looking at how significant it is. By demonstrating how the model outperforms competing deep-learning algorithms, this section emphasizes the model’s promise in otolaryngology and other medical fields.

Radiologykey’s quick search function solves clinical questions quickly.Radiologykey fits everyone.For clinicians, Radiologykey provides the most reliable knowledge and audiovisual across radiological specialties, updated regularly.For Students: Radiologykey supports the most important decisions from classroom to patient bedside.Lecturers: Radiologykey makes it easy for lecturers to find radiological concerns, helping them create the best lectures quickly.Radiologykey customizes to the schedule, procedure, and content demands, rendering it easier to find and use vital data.

The petrous temporal bone contains the air-filled middle ear cavity, often known as the tympanic cavity or tympanum (plural: tympanums/tympana). The tympanic membrane and medial wall separate it from the exterior and inner ears. The three auditory ossicles transport and enhance sound vibrations from the tympanic membrane to the oval window of the inner ear’s lateral wall.

### Results

**Fig. 2:**
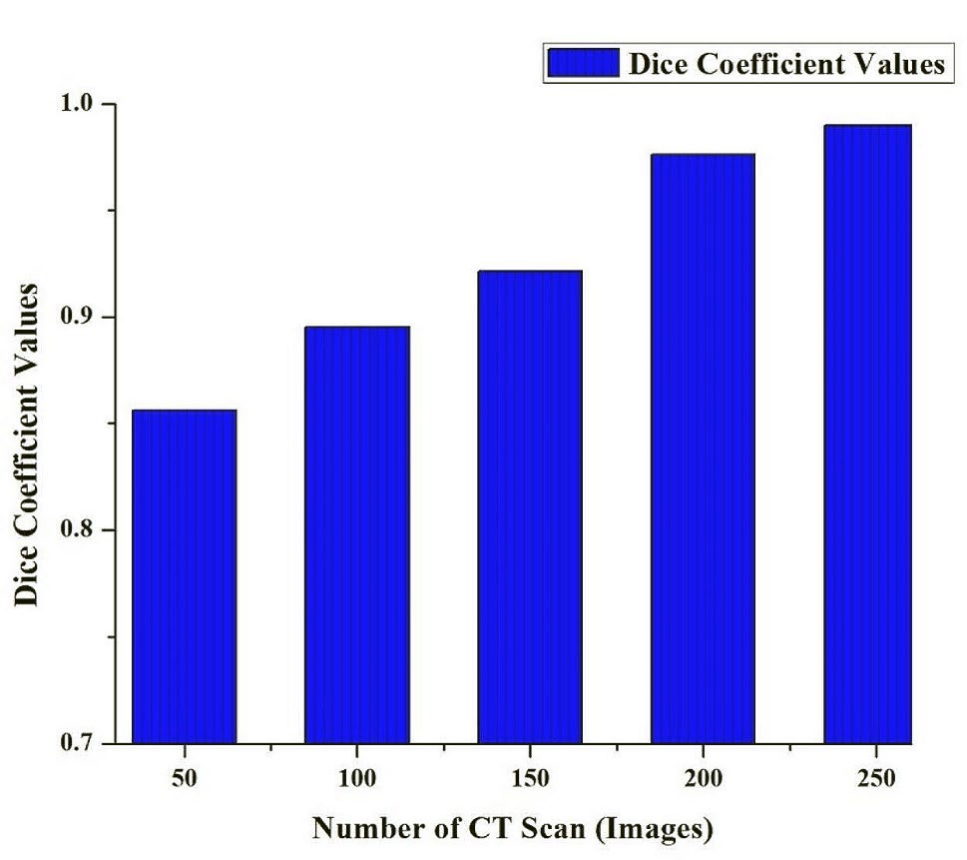
Dice Coefficient Value of the HRSCT-DT Model

The Dice Coefficient, also known as the Srensen-Dice Coefficient, is a critical measure for gauging the segmentation efficacy of the HRSCT-DT model. Values 0 and 1 indicate how much the ground truth mask matches the model’s expected segmentation mask. If the value is 0, there is no spatial overlap; if it’s 1, there is perfect alignment. The higher spatial agreement, as seen by a more significant Dice Coefficient in Fig. 2, indicates the model’s efficacy in segmenting problematic regions across several medical pictures. Otolaryngologists rely heavily on this statistic since it is essential for establishing informed diagnoses and treatment plans. A higher Dice Coefficient suggests better spatial agreement in the context of many processed images.

**Fig. 3:**
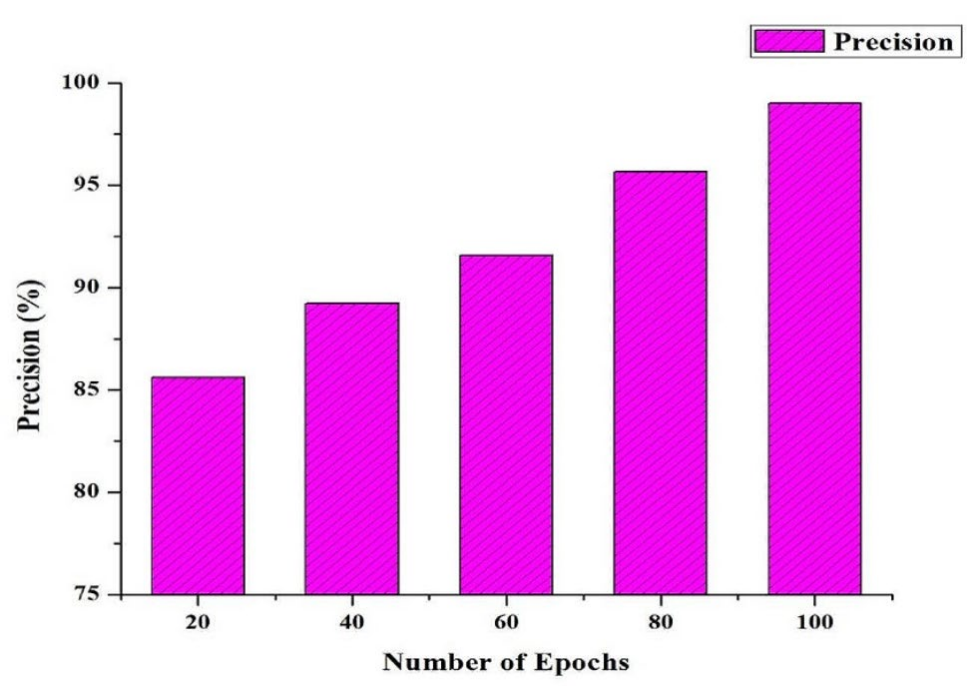
Precision Rate (%) of the HRSCT-DT Model

**Fig. 4:**
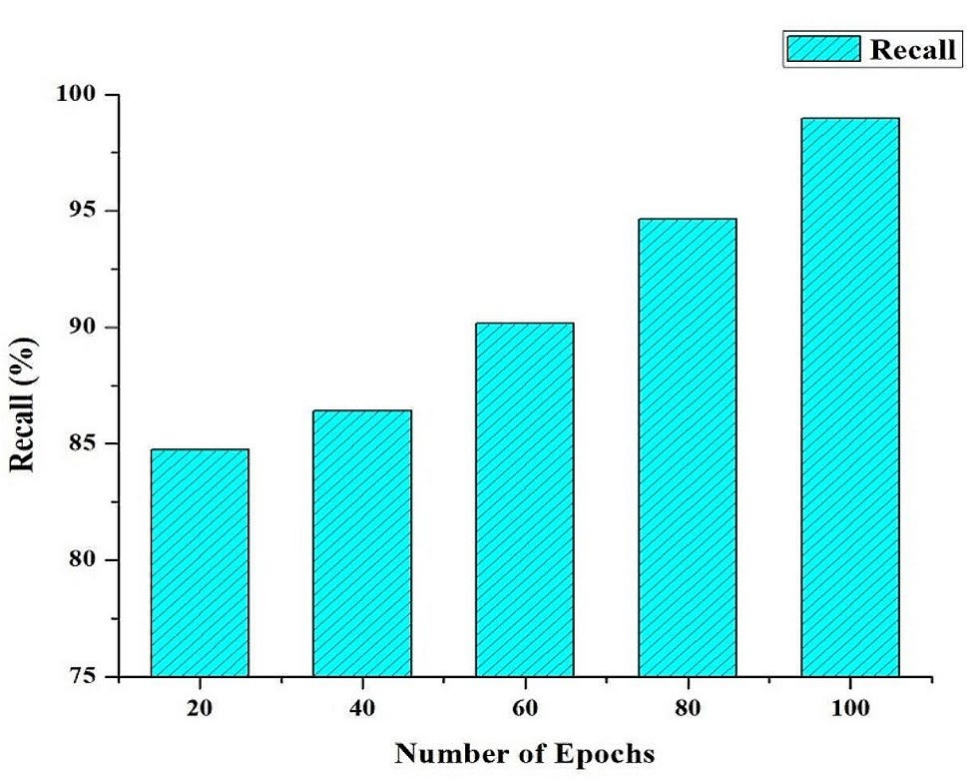
Recall Rate (%) of the HRSCT-DT Model

Precision and Recall emerge as central metrics for evaluating the model’s proficiency in detecting correctly classifying pathological regions during image segmentation tasks (see Figs. 3 and 4 for an illustration of the high-performance HRSCT-DT model’s use of extensive training epochs). Precision measures how accurate the model is at making positive predictions, or “true positives.” A higher Precision score indicates that the model is more likely to predict diseased locations accurately. Recall (sensitivity or true positive rate) measures how well it can spot and include all truly problematic regions when assessing a model’s predictive power. When the Recall score is high, the model is very good at spotting and includes difficult areas of its predictions. Precision and Recall are essential metrics for validating the HRSCT-DT model’s efficacy in identifying and classifying challenging regions using many training epochs. This skill is critical in medical image segmentation, especially in otolaryngology, where a thorough and precise diagnosis is paramount.

**Fig. 5:**
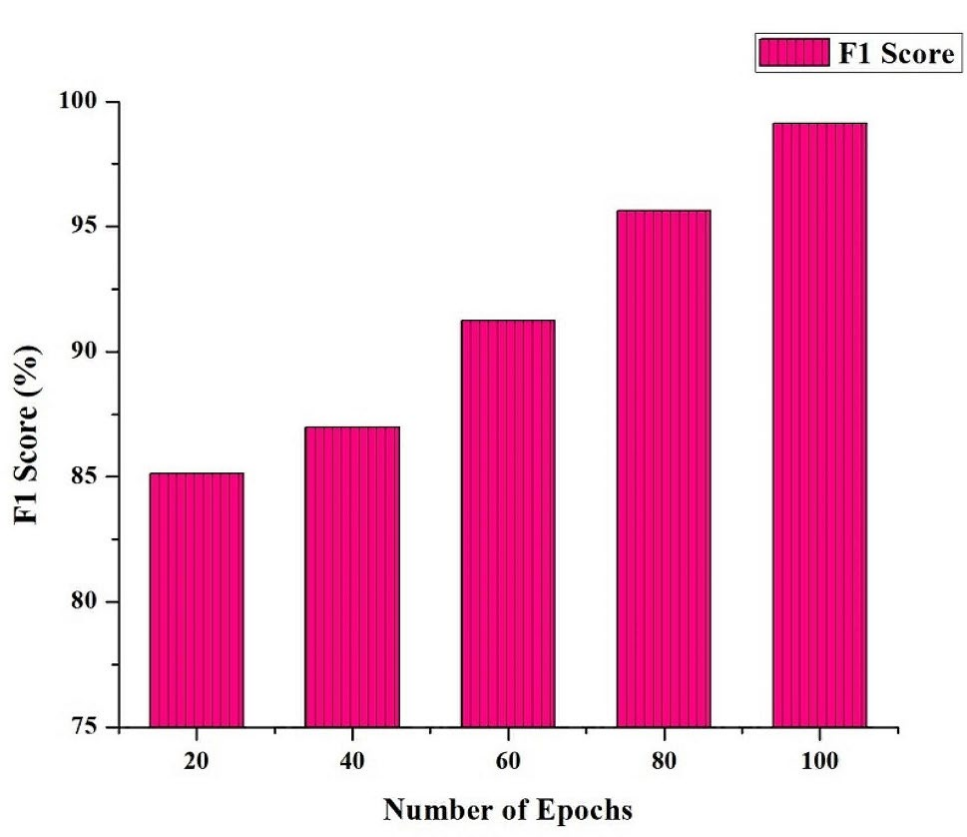
F1 Score (%) of the HRSCT-DT Model

It is essential to recognize a common difficulty in image segmentation, the intrinsic trade-off between precision and Recall, within the effective HRSCT-DT model, which flourishes with many training epochs. Improving one of these indicators could lead to a decline in the other. Therefore, the F1 Score, a helpful indicator, becomes an attractive option. The F1 Score is the harmonic mean of accuracy and Recall, successfully integrating each aspect of model performance. In the context of the HRSCT-DT model, where optimal segmentation is crucial, the F1 Score is an indispensable single metric, harmoniously harmonizing precision and Recall, enabling a full assessment of the model’s performance, as seen in Fig. 5.

**Fig. 6:**
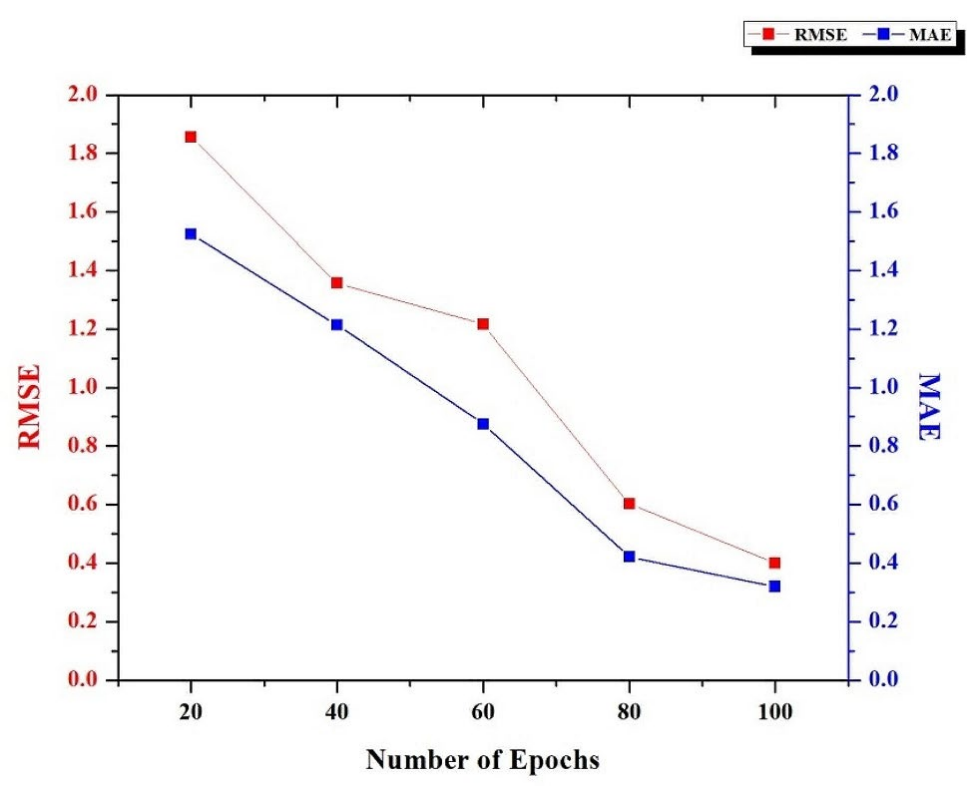
RMSE and MAE Rate of the HRSCT-DT Model

The Intersection over Union (IoU), also known as the Jaccard Index, is a crucial metric in the extraordinary performance of the HRSCT-DT model. As the number of training iterations grows, so does the quality of the results. IoU expertly determines the degree of overlap between the ground-truth regions and the model’s predictions using exact measurements of the intersection and union of the two sets. Figure 6 shows that as the number of training epochs for the HRSCT-DT model increases, the IoU value rises progressively, highlighting the impressive degree to which the predicted and ground truth regions overlap. The IoU value for the HRSCT-DT model steadily increases as the number of training epochs increases, attesting to its superior performance. An IoU of 0 indicates poor segmentation, while an IoU of 1 indicates an exact match. This metric becomes extremely useful when evaluating overlap in intricate segmentations or working with regions of varying shapes.

**Fig. 7:**
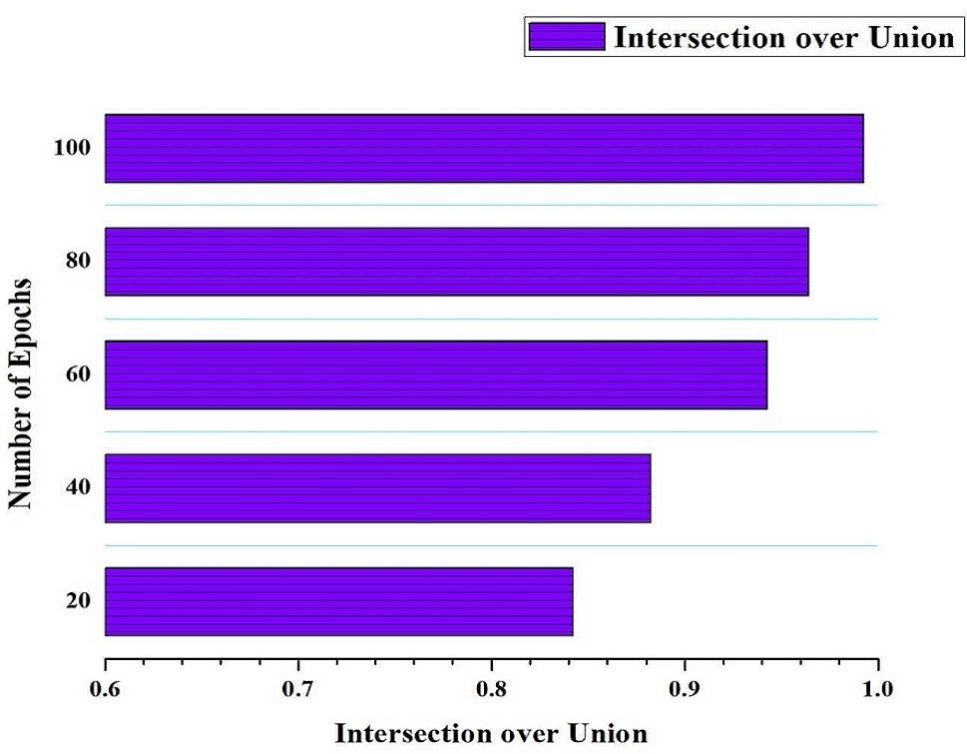
Intersection Over Union Metric of the HRSCT-DT Model

Figure 7 displays the decreased MAE and RMSE values that can be achieved using the HRSCT-DT model as more epochs pass. The mean absolute error (MAE) shows how off the model is, on average, from the actual pixel values. As the number of epochs used in the HRSCT-DT model grows, the MAE score constantly decreases, suggesting an impressively high level of agreement between the predicted and observed values. The RMSE is a more comprehensive measure of the model’s performance. It gives a rough estimate of the forecast error standard deviation. In particular, RMSE’s ability to retain the same units as the pixel values makes it easy to relate to the images’ features directly. In addition, the HRSCT-DT model improves performance with more training iterations.

**Fig. 8:**
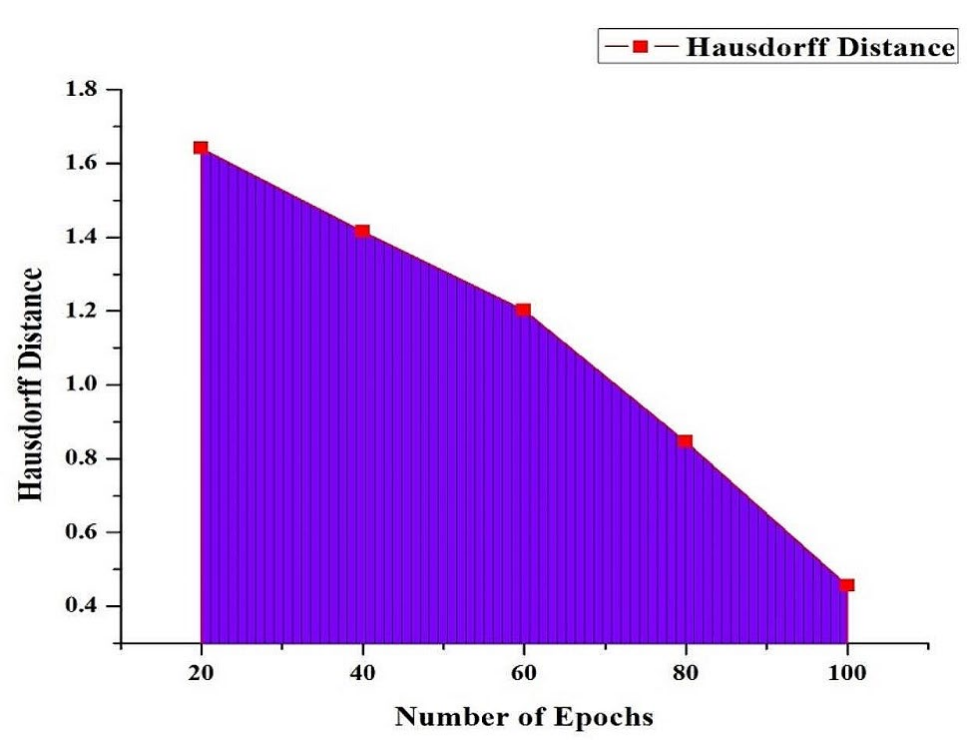
Hausdorff Distance Metric of the HRSCT-DT Model

Figure 8 shows how the Hausdorff distance emerges as a critical metric in the proposed HRSCT-DT model, which offers impressive performance with increased training epochs. This precision distance measure accurately calculates the most significant possible gap between the model’s anticipated and the real-world segmentation borders. The Hausdorff distance within the HRSCT-DT model continually decreases as the number of epochs grows, demonstrating the model’s accuracy. If the projected and ground-truth bounds are similarly near in size, then the model has done an excellent job of delineating the borders.

**Table 3:** Comparative analysis of the HRSCT-DT with other deep learning models

Methods	Precision	Recall	F1-Score	Dice Coefficient	IoU	Diagnostic Accuracy
CNN-GoogLeNet	86.52	85.23	86.45	0.8724	0.8893	0.7496
CNN-DenseNet	89.41	88.47	89.27	0.9006	0.9024	0.7951
CNN-ResNet	92.56	93.64	92.98	0.9421	0.9451	0.8463
Mask R-CNN-UNet	94.27	95.47	94.91	0.9674	0.9689	0.8749
HRSCT-DT	98.01	98.97	99.12	0.9897	0.9924	0.9624

The proposed HRSCT-DT model, created over several training epochs, outperforms competing deep learning models across various metrics (including accuracy, Recall, F1 score, Dice Coefficient, and Intersection over Union; see Table 3). Its recall score is impressive and shows good accuracy in predicting problematic regions. The model achieves a remarkable F1 score by striking a delicate balance between precision and Recall. IoU intensely beats other models in evaluating the degree of intersection between predicted and ground truth regions, and its superior Dice Coefficient illustrates its ability to align these regions precisely. This model excels at analyzing medical images. Examining cost-effectiveness with diagnostic accuracy metrics demonstrates a distinct pattern of enhanced efficacy as more sophisticated models are implemented. The baseline accuracy of CNN-GoogLeNet is 0.7496, followed by CNN-DenseNet at 0.7951 and CNN-ResNet at 0.8463. The Mask R-CNN-UNet model demonstrates a significantly improved accuracy rate of 0.8749. Nevertheless, the HRSCT-DT model reveals the most notable improvement in accuracy, with an outstanding accuracy rate of 0.9624. It implies that although all models exhibit usefulness, the HRSCT-DT model significantly improves diagnosis accuracy, which could lead to improved patient outcomes and cost reductions in healthcare provision.

**Fig. 9:**
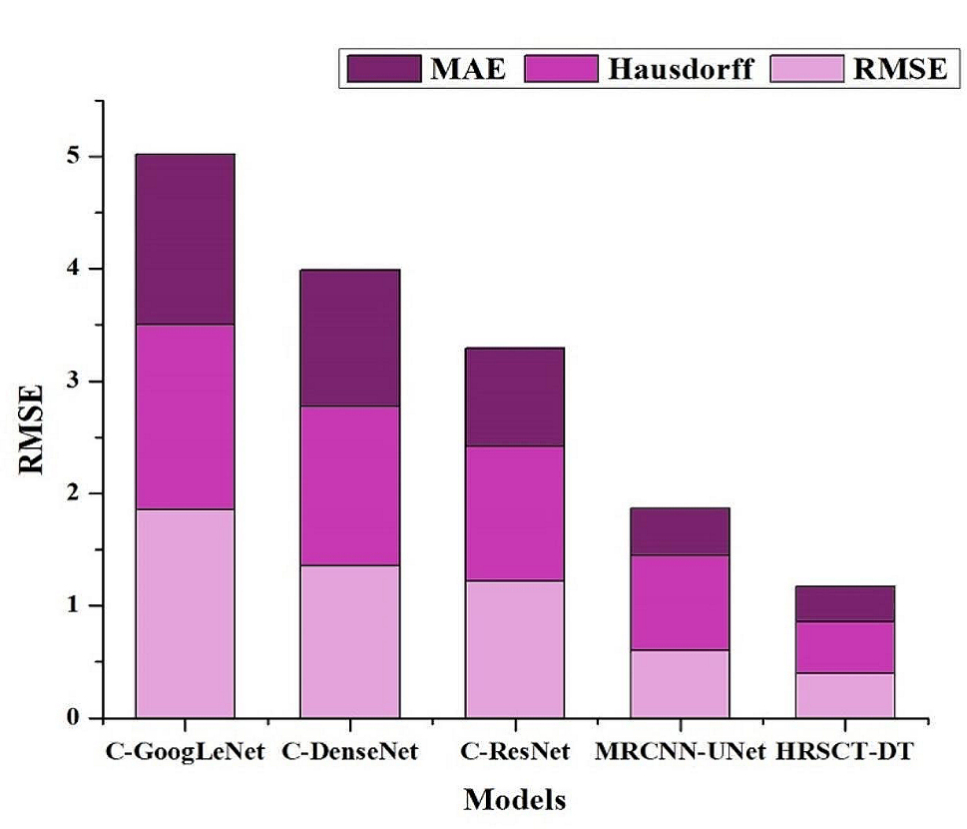
Comparative Analysis of the HRSCT-DT and Other Models with Error Metrics

Figure 9 displays the superior performance of the HRSCT-DT model in medical image analysis using the RMSE, MAE, and Hausdorff Distance Calculation metrics after extensive training. The root-mean-squared error (RMSE) measures how near predicted values are to the real ones. The MAE estimates how far off predictions are from the actual values, with smaller values representing more accurate predictions. Calculating the Hausdorff Distance is a method for determining how near a forecast is to the ground-truth segmentation borders. Accurate border delineation is critical in the processing of medical images.

### Discussion

The suggested HRSCT-DT model is a deep learning model that has undergone rigorous testing and evaluation. The spatial agreement between the predicted and ground truth masks is what the Dice Coefficient uses to determine how well it performs. Accurate diagnosis and clinical decision-making in otolaryngology rely on the model’s steadily rising Dice Coefficient as training epochs accumulate. During effectively detecting and classifying problematic regions, the HRSCT-DT model scores highly on two crucial metrics: Precision and Recall. Its excellent Precision and Recall rates guarantee precise predictions of difficult areas, and its high Recall rate indicates its success in locating and including actual pathological regions of its forecasts. The F1 Score is a comprehensive measure of the model’s efficacy that takes into account the trade-off between accuracy and Recall, a typical challenge in image segmentation. Intersection over Union (IoU) scores highly for the HRSCT-DT model, too, showing an impressive overlap between the model’s predictions and the truth. The model maintains higher IoU values as training epochs grow, demonstrating its superior performance. Predicting pixel values close to the ground truth is essential in medical image analysis, and error metrics like RMSE, MAE, and Hausdorff Distance demonstrate the model’s outstanding accuracy. The model also reflects its precision in border delineation using the Hausdorff Distance measure, which indicates its excellent boundary delineation capabilities. This paper presents a comparative study between the proposed HRSCT-DT model and several existing deep learning models, demonstrating the superiority of the HRSCT-DT model. Compared to competing models, it has superior accuracy, Recall, F1 score, Dice Coefficient, and IoU. The model demonstrates its prowess by accurately highlighting sick spots and properly syncing them with ground truth predictions.

d. It is highly suited for complex segmentations and asymmetrical regions since it can detect sick areas effectively while balancing precision and Recall. The model’s efficacy in predicting outcomes down to the pixel level, as measured by RMSE, also contributes to its usefulness in medical image analysis. The model’s proficiency in delineating boundaries is also evident, with distances increasing smaller and smaller as the number of training epochs increases. Its impressive results suggest it has the potential to greatly improve patient care, especially in areas like otolaryngology, where precise picture segmentation, assessment, and boundary delineation are essential for clinical decision-making and treatment planning.

In this section, we present a comprehensive account of the experiments conducted, the data collected, and the conclusions drawn. It details the experimental framework we developed, the dataset we used, and the way we divided the dataset into training and testing sets. It also describes the evaluation criteria used to evaluate the HRSCT-DT model and provides comparisons to similar models. In this section, we will discuss and assess the findings. In particular, it examines the significance of the efficacy of the HRSCT-DT model and its implications for medical picture analysis. This section highlights the model’s potential in otolaryngology and related medical domains by highlighting how it excels above other deep-learning models.

## Conclusion

This research presents a diagnostic paradigm for otolaryngology incorporating High-Resolution Spiral Computed Tomography scanning and Deep Learning Techniques (HRSCT-DLT). Auriculotemporal and ossicular disorders can be challenging to diagnose, so our project aims to simplify the process for patients and medical professionals. Traditional diagnostic approaches are inadequate for elucidating such diseases. Clinicians and researchers may better capture subtle information within medical pictures thanks to the HRSCT-DLT model’s combination of High-Resolution Spiral Computed Tomography scanning and the CNN-UNet deep learning model. Using automation for essential functions, including ossicle segmentation, fracture diagnosis, and disruption cause categorization, this method can take patient care to new heights and speed up the diagnostic process. Improved diagnosis accuracy and decreased workload for medical professionals are two direct benefits of this automation of clinical decision-making. The HRSCT-DLT model is cutting-edge in medical imaging and diagnostics, giving doctors more tools to make accurate diagnoses and tailor care to each patient. This strategy aims to improve patient outcomes and raise the bar for otolaryngology care overall. High-resolution spiral CT scanning’s radiation exposure, contrast sensitivity, artefact generation, limited functional information, expense, and accessibility are drawbacks. Radiation exposure is hazardous for youngsters and pregnant women, who are more vulnerable. CT doses depend on scan parameters, patient size, and method. CT scans may lack soft tissue contrast, making diseases and soft tissues hard to distinguish. Beam hardening, metal, and motion artefacts can impair image quality and hide key anatomical features or pathology. CT imaging may lack functional or dynamic data, making it less useful for some disorders. High-resolution spiral CT scanners are expensive to buy and maintain, which may limit their use in particular healthcare settings and patient access to diagnostic services. Future research should integrate multimodal imaging methods like MRI and ultrasound with the HRSCT-DLT architecture for a comprehensive diagnostic approach.

This section details the experiments, data, and findings. It describes the experimental methodology, dataset, and training and testing sets. It also outlines the HRSCT-DT model’s evaluation criteria and compares it to others. The HRSCT-DT model excels in medical image segmentation metrics like precision, recall, F1 score, Dice Coefficient, IoU(98.01, 98.97, 99.12, 0.9897, 0.9924), and error measures like RMSE and MAE. It focuses on HRSCT-DT model efficacy and medical picture analysis. The section shows how the model outperforms existing deep-learning models in otolaryngology and related medical fields.

## Data Availability

The data that support the findings of this study are available from the corresponding author upon reasonable request.
